# Requests classification in the customer service area for software companies using machine learning and natural language processing

**DOI:** 10.7717/peerj-cs.1016

**Published:** 2023-03-17

**Authors:** María Ximena Arias-Barahona, Harold Brayan Arteaga-Arteaga, Simón Orozco-Arias, Juan Camilo Flórez-Ruíz, Mario Andrés Valencia-Díaz, Reinel Tabares-Soto

**Affiliations:** 1 Department of Electronics and Automation, Universidad Autónoma de Manizales, Manizales, Caldas, Colombia; 2 Department of Computer Science, Universidad Autónoma de Manizales, Manizales, Caldas, Colombia; 3 Departamento de Sistemas e Informática, Universidad de Caldas, Manizales, Caldas, Colombia; 4 SIGMA Ingeniería S.A, Manizales, Caldas, Colombia

**Keywords:** Natural language processing, Machine learning, Consumer service, Requests classification, Text classification

## Abstract

Artificial intelligence (AI) is one of the components recognized for its potential to transform the way we live today radically. It makes it possible for machines to learn from experience, adjust to new contributions and perform tasks like human beings. The business field is the focus of this research. This article proposes implementing an incident classification model using machine learning (ML) and natural language processing (NLP). The application is for the technical support area in a software development company that currently resolves customer requests manually. Through ML and NLP techniques applied to company data, it is possible to know the category of a request given by the client. It increases customer satisfaction by reviewing historical records to analyze their behavior and correctly provide the expected solution to the incidents presented. Also, this practice would reduce the cost and time spent on relationship management with the potential consumer. This work evaluates different Machine Learning models, such as support vector machine (SVM), Extra Trees, and Random Forest. The SVM algorithm demonstrates the highest accuracy of 98.97% with class balance, hyper-parameter optimization, and pre-processing techniques.

## Introduction

Artificial intelligence (AI) allows the simulation of intelligent human behaviors such as learning and decision making. It is possible due to computational algorithms in the machine learning (ML) field (Da Xu, Lu & Li, 2021). In particular, one of the applications of AI is automatic text classification (TC), which is very useful for characterizing large sets of words from their content (Al-Salemi, Noah & Ab Aziz, 2016). Currently, one of the sectors most benefited from the development of AI is the companies, mainly those with large databases for the implementation of ML techniques (Xu et al., 2020). Brazil with 78%, the United States with 50%, and Mexico with 47% have the highest percentage of companies that apply AI to optimize processes in the American continent (Zemsania, 2021; Reyes, 2020). The objective is to analyze data to obtain more and better business perspectives. However, in Colombia, only 1.8% of companies use AI tools. Applying simple and not very complex AI techniques in Colombia can be a good option for small and medium-sized companies (TigoUne, 2018).

SIGMA Ingeniería S.A is focused on developing Georeferencing software and Geographic Information Systems (GIS) for public management in Colombia as Software as a Service (SaaS). Also, SIGMA Ingeniería S.A is one of the first companies in Colombia to apply AI to solve problems found in the company, specifically those related to customer service. The response times by the technical support area are outside the estimated times for solving the requests (service tickets) made by the clients. The company receives more than 4,000 annual requests, which means 11 daily service tickets presented by its clients, which implies approximately 13,000 hours spent in services in 2020 and 2021. The response times can be optimized if there is a categorization of the needs, which allows establishing a solution path to the technical support area to respond within the estimated time range. The application of AI in the customer service area provides personalized solutions based on machine learning classification techniques. It will enable categorizing the different types of requests submitted by customers (Xu et al., 2020; Raza et al., 2019).

**Figure 1:**
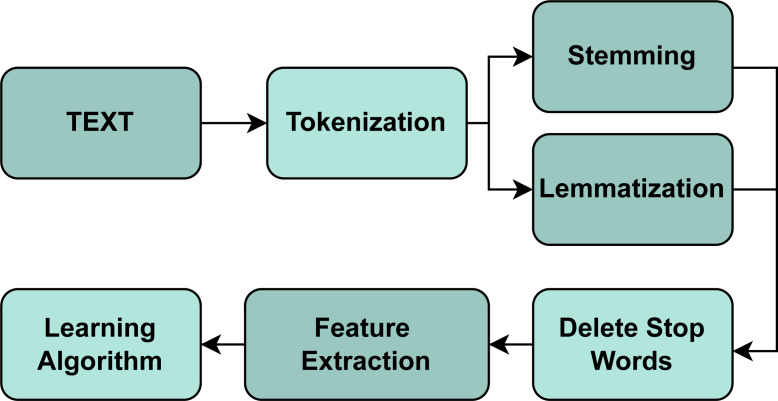
Text classification process.

There is a significant number of studies related to Natural Language Processing(NLP) (Chowdhary, 2020; Singh, 2019; Chopra, Prashar & Sain, 2013; Hirschberg & Manning, 2015). However, Dabrowski et al. (2022) analyzes NLP and data extraction techniques that help develop our work. It performs an application review analysis to examine, transform or model data to discover useful information within them; this is called Content Analysis and allows to find the presence of certain words, themes, or concepts, such as, in our case, the client’s requests. Also, Dabrowski et al. (2022) indicates that the most widely used text mining techniques employ NLP or ML. At this point, text mining and text analysis can allow us to understand syntax (what the word says) and semantics (what the word means) based on information and data obtained by human beings to finally carry out a classification such as TC in this case. In order to perform a good TC process, there are different ways to pre-process the texts, including normalization, cleansing, and augmentation of the text. Gao et al. (2020), Ikonomakis, Kotsiantis & Tampakas (2005) and Kadhim (2019) as shown in the Fig. 1.

Different supervised ML techniques were analyzed for classification problems, such as the NB, SVM, and KNN classifiers. It examines the effect of each technique to classify documents into one or more classes according to their content. The results show that in terms of accuracy, SVM is the best algorithm for all tests using movie reviews on the dataset, and it also tends to be more accurate than other methods. However, it shows that the techniques perform differently depending on the dataset (short and long texts). On this basis, Dharmadhikari, Ingle & Kulkarni (2011) focuses on the existing literature, explores the main classification techniques along with their respective merits and demerits, and explains that the performance of TC algorithms, whether they are supervised, semi-supervised or unsupervised; are highly influenced by the quality of the data source and feature representation techniques since irrelevant and redundant features in the data degrade the accuracy and performance of the classifier. Regarding this study, SVM has been recognized as one of the most effective TC methods (Saravanan & Sujatha, 2018; Wang, Sun & Zhang, 2006) since it can manage large amounts of features and has a high generalization capacity. However, SVM is more complex because it requires more time and memory during the training and classification stages than other supervised learning algorithms (Saravanan & Sujatha, 2018). After considering the text classification as discussed in the previous studies, it is essential to combine it with the multi-label text classification since this work contains a significant amount of labels; in this respect, Wang et al. (2021) designed a reasoning-based algorithm called Multi-Label Reasoner (ML-Reasoner) for the task regarding multi-label classification, in this study they work with techniques such as convolutional neural network (CNN), recurrent neural network (RNN), long Short-Term Memory Network (LSTM), Bidirectional Encoder Representations from Transformers (BERT), binary relevance (BR), classifier chain (CC), label powerset (LP) and other algorithms that have been adapted from SVM. It shows the best results using the LSTM network with 77.60% of precision with 54 different categories in one dataset. Also 91.20% with ML-Reasoner on another dataset with 103 label sets (Wang et al., 2021).

In regard to the studies conducted in this branch focused on support and customer service in companies, Xu et al. (2020) explained that AI in customer service is responsible for providing recommendations, alternatives, and personalized solutions to them. This work exposes an experiment that shows if the consumers of a bank prefer customer service applications with AI or directly with humans. The results show that, in the case of low-complexity tasks, consumers considered that the problem-solving capacity in the AI line was more significant than human customer service. However, they considered that human customer service was superior and more likely to be used than the AI line for high-complexity tasks. On the other hand, Raza et al. (2019) analyzed customer sentiment related to SaaS products. The authors used eleven traditional machine learning classification approaches to accomplish this task. They tested the performance of each of them. This study is essential since they determined the best parameters for each learning algorithm and used an unbalanced data set in terms of distribution of the sample by class, which is very useful to have as a reference in the data to be processed in this work. Furthermore, they have the same characteristics regarding the techniques used, such as NLP, feature extraction using TF-IDF, data balancing with SMOTE, and hyper-parameter optimization application using GridsearchCV from scikit-learn. This study works with ten classes by applying the processes mentioned above; they achieved the best performance with LR with 84.50% accuracy, followed by DT and SVM with 77% accuracy. Lastly, Vera (2017) implemented an automatic classification system for texts of opinions made by clients of a specific company. The system classifies opinions into established categories such as Question, Suggestion, User Information, Company Information, and others. They applied different NLP techniques such as Stop Words removal, lemmatization, and data analytics techniques, including DT, SVM, KNN, and some variants of the NB model. The best results obtained in this study were DT achieving an accuracy of 84.80%, and SVM achieving an accuracy of 84.90% using the TF-IDF representation.

This research seeks to automatically identify and implement ML techniques that characterize the service tickets made by customers in the company SIGMA Ingeniería S.A in 48 categories of the technical support area to reduce the time and complexity to resolve customer requests the systems developed by the company. Previous studies have demonstrated that the best ML algorithm for tests using text datasets and multiple categories is SVM (Kadhim, 2019; Dharmadhikari, Ingle & Kulkarni, 2011; Isa et al., 2008). Considering that this work focuses on understanding text inputs given by customers and company staff, it is necessary to use NLP techniques. It allows us to process text data and understand their meaning and intention (Razno, 2019; Chopra, Prashar & Sain, 2013). The results obtained in the development of the work show that the technique with the highest performance is support vector machine, reaching an accuracy of 98.97% using NLP, balancing techniques and hyper-parameter optimization using GridSearchCV technique through scikit-learn.

This manuscript is organized as follows: “Materials and Methods” section describes the methodology used to apply the three best ML techniques, taking into account the combination of factors such as optimization, and balancing, inbalancing. In “Results” presents the different combinations of factors are arranged to test the given data between hold-out techniques and cross-validation. The “Discussion” provides a detailed analysis of the results obtained and its discussion. Finally, the concluding remarks appear in the “Conclusions” section of the article.

## Materials and methods

In order to characterize the service tickets made by customers based on the best ML techniques, it is necessary to carry out different stages to process the data.

**Figure 2:**
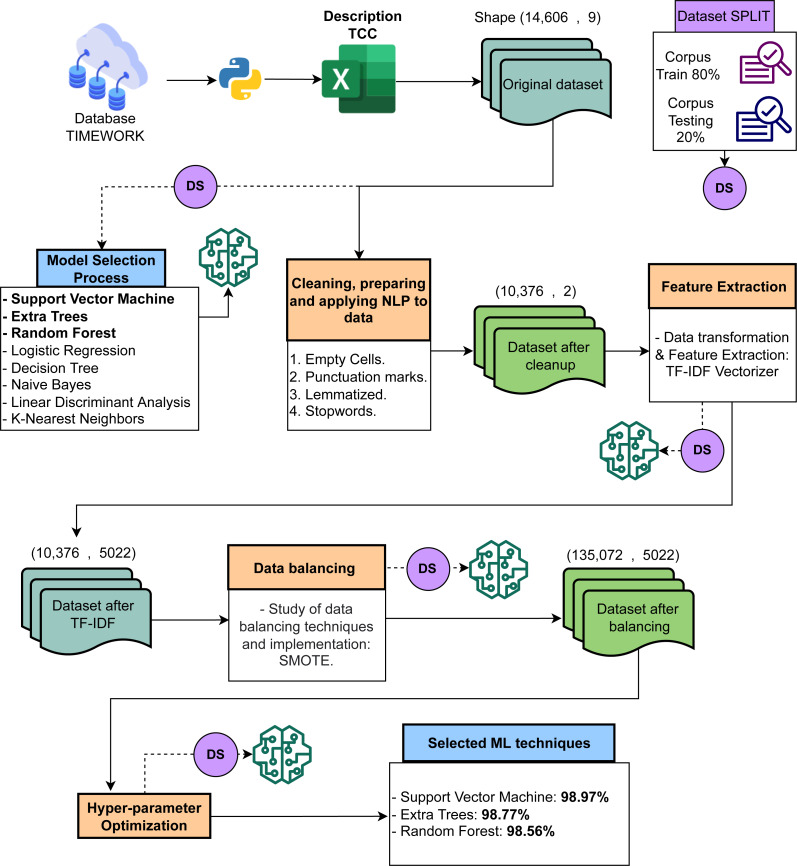
Process conducted for the required text classification.

Figure 2 shows the five main stages of the proposed approach. The stages are described below.

• Reading and interpreting the databases.
• Exploration of eight ML models to identify the best three.
• Pre-processing for cleansing data using NLP techniques.
• Data balancing.
• Generating the highest accuracy percentages using the three best ML techniques.

### Data acquisition

The Timework tool allows knowing, managing, and controlling requests presented by clients. As of May 2021, the Timework platform has approximately 15,000 data or service tickets generated by the organization and work teams. These service tickets describe all the activities of the different methodologies. They are essential to order the management of the company’s internal processes, find patterns and predict behaviors. This research considers 14,606 × 5 data samples, which means 14,606 records with five features such as Ticket code, Description, Category, Customer name, and Business line (See Table 1) that contain information on the requests presented by the clients. This company is from Manizales, Colombia; due to this, all the data it handles and the services offered by the company are in its native language (Spanish). However, for experimentation purposes, the data is translated into English.

In regard to the dataset, Table 2 shows an example of how the Timework platform saves the data internally. This data allows to train and predict with models in the corresponding stage.

Currently, SIGMA Ingeniería S.A company has 48 different categories for classifying the descriptions submitted by the client. Table 3 shows the categories and frequency presented in the databases.

### Data preprocessing

#### Data cleansing

In this first stage of the process, all the empty or null cells of the dataset were removed. It was determined that features such as “Customer name”, “Ticket code”, and “Business line” are fields that do not provide relevant information for the classification process. Experts in the technical support area validate the decision to remove these features. After this cleansing step, the dataset size was reduced from 14,606 × 5 to 10,376 × 2 records.

**Table 1:** Description of fields contained in the dataset (datatype is String for all cases).

**Feature**	Description
Ticket code	Alphanumeric code of the request assigned by the platform.
Description	Description of the request, requirement, or petition submitted by the client.
Category	Internal category to which the description, solicitude, requirement, or request submitted by the client belongs.
Customer name	Customer name associated with the service ticket created.
Business line	Business line on which the request is applied, which can be Geo-lumin, Geo-cleaning, Geo-environmental.

**Table 2:** Some samples of the data for the five features.

**Ticket code**	**Description**	**Category**	**Customer name**	**Business line**
TIK1015	Quality analysis and verifications.	New requirement	Customer 3	Geo-lumin
TIK2942	Error in Widget Add Data - CSV, dll error.	Wrong data in Viewer	Customer 7	Geo-environmental
TIK1577	Customer 4 user manuals	Exchange data for DB	Customer 4	Geo-lumin
TIK2189	Add SE Mosquera project in Geolumina	Exchange data for DB	Customer 3	Geo-lumin
TIK1067	Know the time at which the vehicle that made a route passed	New requirement	Customer 6	Geo-cleaning
TIK2189	Add SE Mosquera project in Geolumina	Exchange data for DB	Customer 3	Geo-lumin
TIK1087	Check shape	Bulk data upload	Customer 10	Geo-cleaning
TIK1690	Customer loads HOME MICRO ROUTES	Bulk data upload	Customer 8	Geo-cleaning
TIK2472	Report V Corpocaldas	New requirement	Customer 15	Geo-environmental
TIK1686	Add status Waiting for Auto Information for profile 08 (legal).	Adding or modifying functionality in profile	Customer 9	Geo-environmental

**Table 3:** Categories contained in the databases.

**Category**	**Frequency**	**Category**	**Frequency**
A mobile user does not log in	18	Exchange data for DB	2814
Adding or modifying functionality in profile	94	Form does not save, does not edit and does not delete	144
Bad data in BI	20	GPS Jumps (Uncalibrated)	101
Bad data in Cube	13	GPS Review	105
Bulk data upload	1150	Generate report, report, data requested by the client	29
Can’t log in to mobile app	15	Implementation of module or new functionality	20
Complete interruption of service	25	Layers don’t load in the viewer	23
Configuration of new Report	187	Missing data in a report	217
Configuration of new widget in viewer	17	Module or functionality training	23
Consultation Review	77	New field setup	24
Creation of users for platform login	45	New requirement	2643
Decreased platform performance	25	Not defined	880
Does not download report	21	Publishing services, layers	24
Does not load the system	22	Shape generation	21
Does not load the viewer	28	Slow viewfinder	17
Does not search or filter in form	13	System audit	22
Does not send the mobile application backup	20	They do not load the data correctly in the application	174
Does not transmit GPS	55	Unable to enter a user	26
Doesn’t load a form	58	Upload mobile application (app) to play store	13
Doubt in platform use	18	Viewer new layer settings	23
Doubt in use of fields	12	Viewer widget not working properly	22
Equipment configuration (GPS)	148	Wrong calculation in form	362
Error appears in mobility when adding	20	Wrong data in Viewer	108
Error exporting report	172	Wrong data in report	268

#### Natural language processing

NLP is a field of AI that helps computers understand, interpret, and manipulate human language. It also investigates how human beings understand and use language to develop adaptation tools and techniques for computer systems to understand and manage natural languages to perform specific tasks (Razno, 2019; Chopra, Prashar & Sain, 2013; Khurana et al., 2017).

There are several techniques for text processing using NLP (Ikonomakis, Kotsiantis & Tampakas, 2005; Sun, Luo & Chen, 2017). In this stage, four of these techniques are implemented:

• Converting texts into lowercase (Uysal & Gunal, 2014; Hadi & Fard, 2020).
• Cleaning the text by removing punctuation marks (Kadhim, 2019).
• Applying text normalization techniques such as stemming or lemmatization (Haroon, 2018; Plisson et al., 2004).
• Removing stop words (Liddy, 2001).

#### Feature extraction

TF-IDF feature extraction was applied to the dataset; this technique can reduce the effect of some common but irrelevant words while retaining important words that affect the whole text. This process is also known as vectorization (Qaiser & Ali, 2018; Ramezan, Warner & Maxwell, 2019).

### Data balancing

Since the database is unbalanced to classify categories, techniques such as Synthetic Minority Oversampling Technique (SMOTE) are applied to solve the class imbalance (Fernández et al., 2018; Han, Wang & Mao, 2005), there are categories with different frequencies, as shown in Table 3, where the category with the lowest frequency is 12, and the highest is 2,814. Therefore, it is necessary to apply balancing techniques that allow homogenizing the distribution of the amount of data by categories (Fung et al., 2006).

Oversampling is the technique for balancing data; this is responsible for creating replicas of the data in the minority classes to reach the amount of data of the majority classes. One of the suitable techniques is SMOTE; this method creates new samples, which is better than up-sampling, and only duplicates existing instances of the minority class. SMOTE synthesizes new samples from the current samples like data augmentation (Fernández et al., 2018; Han, Wang & Mao, 2005). This process has the approval of the company’s experts in the technical support area. They explain that the data to be replicated would adequately simulate the data typically generated in the company’s customer service area when entering a new request or service ticket.

### ML models

The chosen ML techniques that stand out for solving classification tasks are: Logistic regression (LR), linear discriminant Analysis (LDA), decision trees (DT), Random Forests (RF), Extra Trees (ET), k-nearest neighbors (KNN), support vector machines (SVM), and naïve Bayes(NB) (Kotsiantis, Zaharakis & Pintelas, 2006; Osisanwo et al., 2017; Arteaga-Arteaga et al., 2021).

• LR. Logistic regression allows modeling the probability of a given class or a current event. LR also allows working with several classes or more complex events that involve more than two categories (Connelly, 2020).
• LDA. The linear discriminant analysis allows finding a linear combination of features that separates two or more classes of events (Nie et al., 2020). The LDA technique is generally used for dimensionality reduction and data classification (Tharwat et al., 2017).
• DT. This technique classifies by creating a decision tree; this model also allows classification for more than two classes or events (Dumont et al., 2009). DT predicts the value of a dataset by learning a series of decision rules created from the database.
• RF and ET. Both techniques consist of creating multiple decision trees; they are also classification and regression methods; however, overfitting is very likely to occur with the training data and avoid this problem. RF is in charge of training several decision trees. The output is the class that most of the trees have selected (Géron, 2019; Breiman, 2001). On the other hand, the ET technique provides more randomness to the training set by using different decision thresholds for each input element (Geurts, Ernst & Wehenkel, 2006).
• KNN. It is a classification technique that is used for classification and regression. KNN is responsible for classifying data by evaluating the distance between neighboring data. When evaluating a sample, KNN assigns weights to all neighboring points and performs classification by looking for the closest points learned in the training stage. Then it chooses the most frequent class (Guo et al., 2003).
• SVM. This technique is also used for classification and regression. SVM provides better results when working with small and medium data sets. The primary function of the technique is to separate classes or events as far as possible from the decision boundary of the closest training samples (Géron, 2019; Gholami & Fakhari, 2017). SVM has been recognized as one of the most effective TC methods (Haryanto, Mawardi & Muljono, 2018; Wang, Sun & Zhang, 2006; Vijayan, Bindu & Parameswaran, 2017).
• NB. This classifier uses Bayes’ theorem (Perez, Larranaga & Inza, 2006). In addition, NB assumes that a specific characteristic’s effect on a category is independent of other classes. In other words, each data can have a probability of belonging to a specific data set without other samples.

The ML techniques will be conducted through Hold-out methods and cross-validation to provide more scope for understanding the final results.

### Performance assessment

Evaluating machine learning algorithms is an essential part of any project. The model may perform well when evaluated against one metric but poorly assessed against other metrics (Hossin & Sulaiman, 2015; Arteaga-Arteaga et al., 2021).

• **Accuracy**: is the total number of input samples ratio to the number of correct predictions (Hossin & Sulaiman, 2015; Arteaga-Arteaga et al., 2021):
  (1) \begin{eqnarray*}Accuracy= \frac{TP+TN}{TP+TN+FP+FN} \end{eqnarray*}
• **Precision**: this metric allows for determining the number of correct positive predictions made (Hossin & Sulaiman, 2015; Arteaga-Arteaga et al., 2021).
  (2) \begin{eqnarray*}Precision= \frac{TP}{TP+FP} \end{eqnarray*}
• **Recall**: this metric is used to determine the number of correct positive predictions made between the total number of positive predictions made (Hossin & Sulaiman, 2015; Arteaga-Arteaga et al., 2021).
  (3) \begin{eqnarray*}Recall= \frac{TP}{TP+FN} \end{eqnarray*}
• **F1-score**: is the Harmonic Mean between accuracy and recovery (Hossin & Sulaiman, 2015; Arteaga-Arteaga et al., 2021). The higher the F1 score, the better the performance of the model:
  (4) \begin{eqnarray*}F1-score=2\times \frac{Precision\times Recall}{Precision+Recall} \end{eqnarray*}
• **Classification Report**: The classification report visualizer builds a text report showing the main classification metrics such as precision, recall, F1-score, and *support* is the number of occurrences of the given class in the dataset (Hossin & Sulaiman, 2015; Arteaga-Arteaga et al., 2021).
• **Confusion Matrix**: A confusion matrix summarizes the number of predictions made by a model for each class and the classes to which those predictions belong. It helps to understand the types of prediction errors made by a model (Hossin & Sulaiman, 2015; Arteaga-Arteaga et al., 2021).

### Resources

All algorithms exposed in this work are developed and executed using Python 3.8. The machine used has Windows 11 (64-bits) as the operating system and an AMD Ryzen 7 5800H processor, RAM of 16.0GB, and Radeon Graphics 3.20 GHz. The code and data are in a GitHub repository, as shown in the “Data Availability” section.

## Results

The results are shown for four experiments designed; these tests consist on different combinations regarding pre-processing, balancing, and ML algorithms with hyper-parameter optimization (using GridSearchCV of scikit-learn) (Weerts, Mueller & Vanschoren, 2020; Batista, Prati & Monard, 2004; Demidova & Klyueva, 2017). In this way, four experiments were defined as follows: the Original Dataset (OD), a Dataset with Pre-processing (DP), a Dataset with Pre-processing and Balancing (DPB), a Dataset with Pre-processing, Balancing, and Optimization (DPBO) (see Table 4).

• OD. For this experiment, the data set is taken in raw, no pre-processing is made to it, and the proposed technique is applied exactly as it is.
• DP. In this case, some pre-processing is applied to the original dataset, such as NLP techniques and feature extraction through TF-IDF Vectorizer explained in “Natural Language Processing“ and “Feature extraction” previously.
• DPB. For this experiment, all the techniques applied in DP, as well as data balancing methods, are conducted.
• DPBO. This last experiment applies all the techniques mentioned in DPB. Also, it applies hyper-parameter optimization through the chosen machine learning techniques.

The experiments have two data partitions (Hold-out), training with an 80% of data and the remaining 20% for testing (Feurer et al., 2018). Also, the results have cross-validation with 10-folds; it allows seeing the most stable ML algorithm in its average accuracy and standard deviation.

In this way, the first test performed on the data was through eight ML techniques explained in the “ML Models” subsection. These results show the performance obtained by the ML techniques when working with the OD, without preprocessing, without balancing, and without hyper-parameter tuning (see Table 5), where the best three models (SVM (64.07%), ET (63.87%), and RF (64.26%)) are selected to execute the remaining experiments shown in Table 4.

**Table 4:** Textual indicators assigned to the tests performed.

**Experiment setup**	**Abbreviation**
Original Dataset	OD
Dataset with Preprocessing	DP
Dataset with Preprocessing and Balancing	DPB
Dataset with Preprocessing, Balancing and Optimization	DPBO

### Hold-out

After selecting the best three ML models, the Hold-out process is conducted for each experiment (OD, DP, DPB, DPBO). Table 6 shows the first results when applying the proposed methodology. However, SVM reaches the best performance among all the evaluated techniques. A significant difference is shown between OD and DP experiments compared to DPB and DPBO experiments; the main difference between these groups is the balance methods applied. In addition, it is shown that making hyper-parameter tuning is an effective method to increase the ML techniques’ performance further.

**Table 5:** Comparison of the accuracy obtained between the eight ML algorithms initially selected. The bold entries indicate the best results.

**ML technique**	**Accuracy [%]**
Support Vector Machine	**64.07**
Extra Trees	**63.87**
Random Forest	**64.26**
Logistic Regression	62.28
Decision Trees	56.26
Linear Discriminant Analysis	57.27
Naive Bayes	48.55
KNeighbors	44.17

**Table 6:** Comparison of the scores obtained between the selected algorithms (SVM, RF, and ET) with the proposed conditions. The bold entries indicate the results of the best algorithm for the BPBO scenario.

**Experiment**	**ML technique**	**Accuracy [%]**	**F1-Score [%]**	**Recall [%]**	**Precision [%]**	Training time [Sec]	**Prediction time [Sec]**
OD	SVM	64.07	58.55	64.07	62.31	14.664	1.634
	ET	64.35	59.68	64.35	61.04	7.612	0.109
	RF	64.11	59.47	64.11	61.29	5.997	0.078
DP	SVM	64.69	59.48	64.69	62.78	14.593	1.6389
	ET	63.87	59.40	63.78	61.35	7.339	0.093
	RF	64.31	59.65	64.31	61.15	5.755	0.078
DPB	SVM	98.76	98.74	98.76	98.75	143.929	86.613
	ET	98.64	98.60	98.64	98.60	137.140	1.316
	RF	98.42	98.40	98.42	98.41	99.558	1.038
**DPBO**	**SVM**	**98.97**	**98.95**	**98.97**	**98.95**	**116.982**	**72.499**
	ET	98.77	98.74	98.77	98.83	132.655	1.320
	RF	98.56	98.53	98.56	98.52	783.018	7.847

To better evaluate the best ML model (SVM), the metrics F1-score, recall, and precision allow a better understanding of the achieved results. Table 6 also shows the performance of the classifiers and the time spent by each technique in each experiment during the training stage.

After testing all these traditional ML techniques we decided to compare the results through other techniques using the DPBO experiment because the best results were obtained in this test. Other techniques such as GPT3 (Olmo, Sreedharan & Kambhampati, 2021) and deep learning models using CNN (Feng & Cheng, 2021; Malekzadeh et al., 2021) were tested with the dataset (see Table 7). However, the results were not as satisfactory as the traditional ML techniques (SVM), for this reason we continue using the techniques shown in Table 6).

Some methods such as Bag of Words with TF-IDF vectorizer and Count vectorizer (Dessi et al., 2021; Grootendorst, 2022), word Embedding with Word2Vec (Di Gennaro, Buonanno & Palmieri, 2021; Dessi et al., 2021), and the cutting edge language models with BERT (Prabhu, Mohamed & Misra, 2021; Grootendorst, 2022) were also tested with the dataset. The best results were obtained with TF-IDF technique due to the computational reduction time, dimensional issues, and simplicity of its implementation. Table 8 shows the results obtained by applying the above techniques.

**Table 7:** Comparison between the best model presented in our work (SVM) (in bold )and other pioneering classification methodologies in TC such as GPT3, and CNN methods.

**Technique**	**Accuracy [%]**
**SVM (our work)**	**98.87**
GPT3	94.04
CNN	90.70

**Table 8:** Comparison between the best technique presented in our work (TD-IDF) (in bold) and other pioneering TC vectorization methodologies such as Word2Vec and BERT.

**Technique**	**Accuracy [%]**
**TD-IDF (our work)**	**98.87**
Word2Vec	93.84
BERT	92.01

Table 9 compares the results obtained for each metric using the Spanish and English languages in the databases. The difference between times and performances is very similar between them, which allows generalizing the study and use the English language to facilitate the reproducibility of the work.

**Table 9:** Comparison of the scores obtained between using English and Spanish language in the databases with the SVM technique in DPBO experiment.

**SVM - DPBO Experiment**							
**Language**	**ML technique**	**Accuracy [%]**	**F1-Score [%]**	**Recall [%]**	**Precision [%]**	**Training** time [Sec]	**Prediction time [Sec]**
**Spanish**	**SVM**	98.95	98.93	98.95	98.94	127.718	76.730
	**ET**	98.63	98.60	98.63	98.60	141.338	1.376
	**RF**	98.50	98.48	98.50	98.48	849.049	8.631
**English**	**SVM**	98.97	98.95	98.97	98.95	116.982	72.499
	**ET**	98.77	98.74	98.77	98.83	132.655	1.320
	**RF**	98.56	98.53	98.56	98.83	783.018	7.847

**Figure 3:**
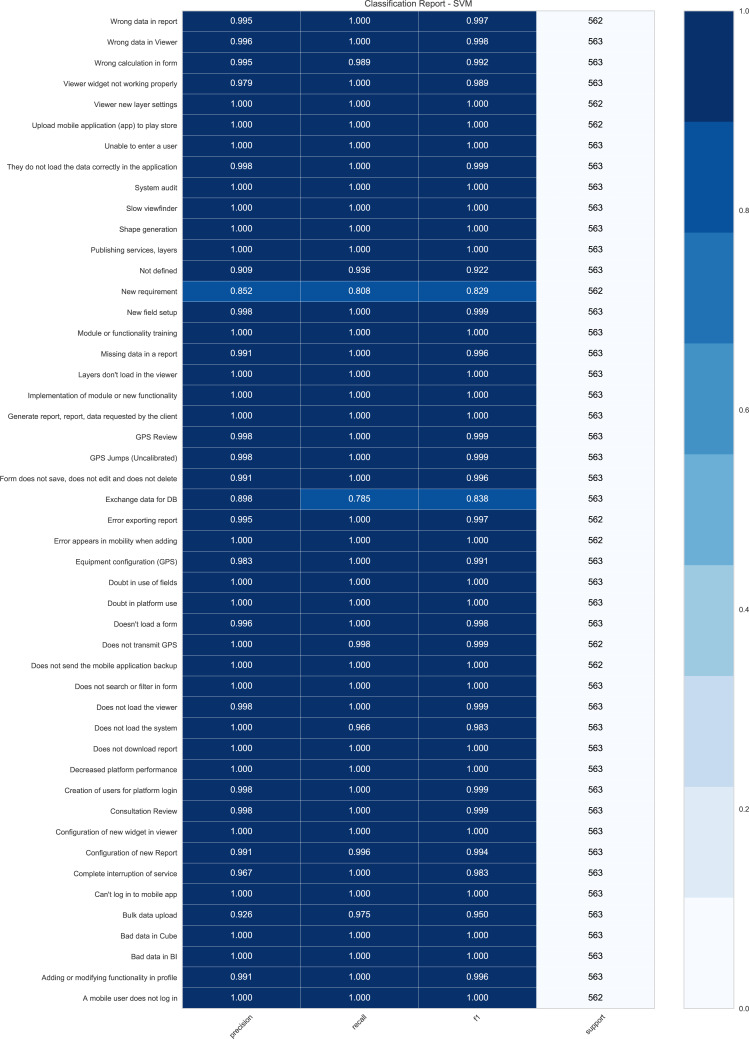
Report Classification for SVM in DPBO (you can apply zoom in as you desired).

Regarding the results obtained through the SVM technique in each test, it is useful to visualize metrics such as Report Classification Fig. 3. This figure presents the Classification Report of the DPBO test for the SVM technique that indicates the precision, recall, F1-score, and support obtained for each class contained in the data set.

It is evident that the results obtained for each of the categories are very high; all of them are found in a percentage above 85.2% with an average of 98.83% for precision, 78.5% with an average of 96.86% for recall, and 82.9% with an average of 98.83% for F1-score. This implies an outstanding performance on the SVM model for classifying the requests in each category. This demonstrates how well the SVM model works for the classification of each class.

### Cross validation

This work presents Cross-Validation with 10-folds, this process is also conducted for each experiment (OD, DP, DPB, DPBO). The results of each experiment are presented in Table 10, where we report the accuracy achieved, followed by the corresponding standard deviation.

**Table 10:** Comparison of the accuracy obtained by Cross-Validation applied to the selected techniques with the established conditions. The bold entries indicate the results of the best algorithm for the BPBO scenario.

**Experiment**	**ML technique**	**Cross valid [%]**	**Time [Sec]**
OD	SVM	63.55 ± 1.53	28.019
	ET	63.53 ± 1.67	14.642
	RF	62.99 ± 1.77	10.865
DP	SVM	64.11 ± 1.82	26.557
	ET	64.62 ± 1.76	13.666
	RF	63.84 ± 1.86	10.536
DPB	SVM	98.91 ± 0.09	534.502
	ET	98.79 ± 0.06	324.263
	RF	98.57 ± 0.11	214.119
**DPBO**	**SVM**	**98.98 ± 0.07**	**440.990**
	ET	98.77 ± 0.06	318.139
	RF	98.60 ± 0.08	1,646.039

## Discussion

The design of the classification system consisted of six steps: data cleansing, application of NLP techniques, feature extraction, balancing, partitioning of data sets, and evaluation of metrics. First, cleansing the dataset, applying NLP techniques, and feature extraction are essential data preprocessing steps when working with textual data. Data balancing is proper when the dataset is unbalanced in large proportions, which would alter the data partition between training and testing and would not provide very reliable results. It is essential to find appropriate data partition sizes that allow the model to generalize well in the training process and demonstrate reliability when tested. The training set represents 80% of the total samples, and the test set the remaining 20%.

In the test performed on Table 8 with the Bag of Words method, TF-IDF is better for this work because Count Vectorizer only focuses on the frequency of the words presented in the dataset, on the other hand TF-IDF additionally provides the word importance which is very useful for this study. Regarding Table 6, the scores increases for DPB and DPBO concerning DP shows that the balancing techniques provide better accuracy results. The explanation is that it occurs when there is a difference of 2, 802 units between the frequency of the highest and lowest category (Fernández et al., 2018). Regarding parameter optimization, this tends to increase for SVM and ET with 98.97% and 98.77% respectively, obtaining SVM as the highest performance technique for problems such as text classification in this case as shown in Table 6 (Ramezan, Warner & Maxwell, 2019).

The results obtained in Table 9 shows that either using the Spanish or English languages in the databases, the results are very similar to each other. Nevertheless, the English language was finally chosen to generalize and facilitate the reproducibility of the work.

Figure 3 shows promising results for all categories except for categories such as “New Requirement“ and “Exchange data for DB.” These classes mentioned above represent approximately 74.59% of errors because the classes are very similar to others, such as “Not defined”. Therefore, the erroneous predictions represent 1.13% of all predictions made (307 errors in 27, 015 predictions). According to the technical support team, some categories are not assigned to specific requests within the company and are very general and confusing among them. Therefore, it was proposed to SIGMA Ingeniería S.A to replace or delete these categories that are not directly related to a specific requirement or request. On the contrary, most categories have a high number of correct predictions. Moreover, according to the technical support area, these categories contain proper keywords for predictions based on the previous training. Therefore, 95.8% of all the classes evaluated have an average precision of 99.33% in the predictions.

The accuracies and standard deviations of the experiments carried out are shown in Table 10. Through this method, the SVM technique has the best performance again. The techniques’ performance change through the application of parameter optimization and data balancing is small, with the “DPBO” option being 0.09% better than the “DPB” option (Komer, Bergstra & Eliasmith, 2014).

Table 11 shows the current literature on multi-class text classification regarding customer service shows that the performance of different text classification algorithms depends on the nature of the textual data and the number of different categories used. Apart from this, the performance of these multi-class text classification models is also affected by other aspects of the dataset, such as imbalanced class distribution, data cleansing, NLP techniques, and many features. Therefore, our work obtained the best results with more categories than other studies (Wang et al., 2021; Raza et al., 2019; Vera, 2017).

**Table 11:** Comparison of multi-class text classification problems approaches regarding customer service.

	**Our work**	**(Wang et al., 2021)**	**(Raza et al., 2019)**	**(Vera, 2017)**
**# of categories**	48	54	10	5
**Best accuracy**	98.97%	77.60% (Precision)	84.50%	97.00%
**Best technique**	SVM	LSTM	LR	DT
**ML techniques used**	SVM	BR	KNN	
	RF	CC	NB	
	ET	LP	DT	DT
	LR	CNN-RNN	SVM	SVM
	DT	LSAN	LR	KNN
	LDA	LSTM	Rigde	NB
	NB	BERT	Perceptron	
	KNN	ML-Reasoner	SGD	
**NLP techniques used**	Data cleansing			TF-IDF
	Stop words removal		Data cleansing	TR-RFL
	TD-IDF	Text Encoder	Bag of words	Stop words removal
	Lemmatization	Text Decoder	TF-IDF	Lemmatization
	Balancing Techniques (SMOTE)		Balancing Techniques (SMOTE)	PCA

Finally, the SVM algorithm generates the best performance for the text classification problem for the company SIGMA Ingeniería S.A. The best accuracy is 98.97%, and 98.98 ± 0.07% using cross-validation. However, ET and SVM do not have significant differences between the accuracies (0.20%), notwithstanding SVM shows the best performance of this type on text classification.

## Conclusions

Three of the eight traditional machine learning approaches were applied to automate category classification on customer support issues regarding the requests presented by the clients in SIGMA Ingeniería S.A, which currently handles customer support requests manually. The company has 48 categories representing an individual quality aspect in the technical support area. What was achieved in this work was to automatically classify the tickets of the support area, immediately improving the attention time and the solution to customer problems. Furthermore, as our dataset faced the problem of class imbalance, evaluation metrics such as accuracy, precision, recall, F1-score, and classification report were implemented to evaluate the performance of each classification model. the results obtained suggest that the Support Vector Machine model achieves the highest performance with an accuracy of 98.97% previously applying the dataset techniques of NLP, hyper-parameter optimization, data pre-processing, and balancing, the latter being the most relevant to achieve such precision. This computational model allows transferring the predicted category to the technical support area, facilitating the identification of the solution protocol to provide a response to the user within the estimated time by the company’s customer service team.

As future work, it is desired to implement an automatic solution protocol system based on AI. On this wise, the model provides the category to which the request presented belongs and provides the solution protocol to be carried out by the technical support area. Accordingly, the precision in the solution of the requirement increases, response times are reduced, and customer satisfaction increases. Additionally, the data generated during the pilot test at SIGMA Ingeniería S.A can be used to further train and improve the SVM algorithm.

## Additional Information and Declarations

### Data Availability

The following information was supplied regarding data availability: The code and data are available at GitHub: https://github.com/BioAITeam/Requests-classification-using-ML-and-NLP and Zenodo: María Ximena Arias-Barahona, Harold Brayan Arteaga-Arteaga, Simon Orozco-Arias, Juan Camilo Flórez-Ruíz, Mario Andrés Valencia-Díaz, & Reinel Tabares-Soto. (2022). Requests classification in the customer service area for software companies using Machine Learning and Natural Language Processing. Peerj Computer Science. https://doi.org/10.5281/zenodo.6578685.
